# Strength, Multijoint Coordination, and Sensorimotor Processing Are Independent Contributors to Overall Balance Ability

**DOI:** 10.1155/2015/561243

**Published:** 2015-11-19

**Authors:** Emily L. Lawrence, Guilherme M. Cesar, Martha R. Bromfield, Richard Peterson, Francisco J. Valero-Cuevas, Susan M. Sigward

**Affiliations:** ^1^Department of Biomedical Engineering, University of Southern California, Los Angeles, CA 90089, USA; ^2^Division of Biokinesiology and Physical Therapy, University of Southern California, Los Angeles, CA 90033, USA

## Abstract

For young adults, balance is essential for participation in physical activities but is often disrupted following lower extremity injury. Clinical outcome measures such as single limb balance (SLB), Y-balance (YBT), and the single limb hop and balance (SLHB) tests are commonly used to quantify balance ability following injury. Given the varying demands across tasks, it is likely that such outcome measures provide useful, although task-specific, information. But the extent to which they are independent and contribute to understanding the multiple contributors to balance is not clear. Therefore, the purpose of this study was to investigate the associations among these measures as they relate to the different contributors to balance. Thirty-seven recreationally active young adults completed measures including Vertical Jump, YBT, SLB, SLHB, and the new Lower Extremity Dexterity test. Principal components analysis revealed that these outcome measures could be thought of as quantifying the strength, multijoint coordination, and sensorimotor processing contributors to balance. Our results challenge the practice of using a single outcome measure to quantify the naturally multidimensional mechanisms for everyday functions such as balance. This multidimensional approach to, and interpretation of, multiple contributors to balance may lead to more effective, specialized training and rehabilitation regimens.

## 1. Introduction

It is well known that both sensory and motor systems contribute to the ability to maintain balance. Sensory inputs are necessary to detect unstable conditions (i.e., perturbations to the system) and motor contributions are vital to initiate timely and appropriate responses to counteract these perturbations. Clinical outcome measures such as single limb balance (SLB), Y-balance (YBT), and the single limb hop and balance (SLHB) tests are commonly used to quantify balance in individuals when they are healthy [1–4] or following musculoskeletal injury (e.g., ankle sprains and anterior cruciate ligament (ACL) tears) [5–11] or to assess risk for lower extremity injury [6, 12–14]. Results obtained from these tests are used to represent the mechanisms of balance. However, the contributions of sensory inputs and appropriate motor responses necessary to perform well vary across them. Outcome measures that include smaller changes in lower limb or whole-body position are typically considered measures of static stability of balance, whereas measures that include larger changes in position are often referred to as dynamic stability of balance. One may argue that detection of smaller changes in position or motion would be more challenging for the sensory system to detect and less challenging for the motor system to counteract; conversely, large changes in position or motion would be more easily detected by the sensory system and, in turn, place greater demands on the motor system to counteract in terms of strength and multijoint coordination. As a result, interpretation of the outcomes with respect to underlying sensory or motor deficits becomes challenging when considering the range of static and dynamic measures used to quantify balance.

Unperturbed single limb balance during quiet standing balance tests generally result in relatively small joint excursions and are considered measures of static balance. This requires detection of smaller, subtler sensory stimuli and relatively small motor responses to maintain balance. In contrast, successful performance on balance tests such as the single limb hop and balance and Y-balance tests involve larger changes in position and are considered measures of dynamic balance. The SLHB quantifies the ability to stabilize the center of mass (COM) after completing a forward hop on a single limb. The transition from a dynamic to a static state can be considered a perturbation to the COM, thus making it a measure of dynamic balance. Performance of both SLB and SLHB is quantified using outcome measures related to center of pressure (COP) movement because they represent corrective actions made to maintain balance [15]. Additionally, performance of the YBT is scored by measuring the farthest distance reached with the free limb while maintaining balance on the stance limb. The maximal reach distances in each of the three directions are considered measures of dynamic balance because changing the spatial orientation of the free limb acts as a perturbation to the COM with respect to the base of support (BOS), or stance limb. For more dynamic tests, while detection of larger joint excursions may be less challenging to the sensory system they also require greater motor responses with respect to lower extremity strength and multijoint coordination [2, 16]. Accordingly, positive correlations between lower extremity strength and performance during these tests suggest that the ability to detect underlying sensorimotor deficits may be limited during these more dynamic tasks [2, 17].

While balance tests are thought to provide insight into sensorimotor processing, it is difficult to test these mechanisms in isolation during traditional balance tests. Therefore, we introduce the Lower Extremity Dexterity (LED) test, which has been proven to quantify sensorimotor processing to control instabilities while controlling for the confounding factors of strength and whole-body equilibrium [18, 19]. The test is based on the principles of the upper extremity Strength-Dexterity (SD) test, which is a repeatable and informative paradigm that has successfully quantified differences in finger dexterity attributed to age, sex, and numerous clinical impairments [18, 20–23]. The SD test quantifies sensorimotor processing for dynamic finger function because it is independent of strength [21, 24] and engages distinct cortico-striatal-cerebellar networks in a context-sensitive way [25, 26]. Building on this paradigm, the LED test quantifies the ability of the isolated lower limb to dynamically stabilize an unstable interface with the ground by controlling the force vectors and motions of the foot [18, 19]. Performance of the LED test is a measure of lower extremity sensorimotor processing that is independent of strength [21], predictive of agility performance in soccer athletes [27], and informative of age- and sex-related effects [18, 28]. Understanding the relationships between LED test and clinical outcome measures can provide insight into the sensitivity of these measures for detecting sensorimotor deficits. Moreover, considering the LED test together with outcome measures will help elucidate how sensorimotor processing contributes to balance.

It stands to reason that balance likely requires a combination of strength, multijoint coordination, and sensorimotor processing that are quantified to varying degrees using numerous outcome measures, several of which are described above. Given the varying demands across tests, it is likely that traditional balance tests provide useful, although test-specific, information regarding the contributors to balance. However, the extent to which these factors contribute to balance and how these outcome measures relate to them are not clear. Therefore, the purpose of this study was to determine the relationships and hierarchy among these outcome measures for balance, strength, and sensorimotor processing in healthy and active young adults.

## 2. Materials and Methods

Thirty-seven young adults (18 F, 19 M) between the ages of 18 and 30 years (mean ± standard deviation; age: 24.7 ± 2.7 yrs; body mass: 74.4 ± 14.2 kg; height: 1.8 ± 0.1 m) and engaged in recreational sports activities agreed to participate in this study. Participants were excluded if they had (1) any lower extremity injury or surgery within the last 12 months, (2) a current upper or lower extremity injury with persistent pain and/or inability to fully participate in sport, (3) a concurrent pathology or morphology that can cause pain or discomfort during physical activity, or (4) any physical, cognitive, or other condition that would impair their ability to perform the tasks proposed in this study. Prior to participation, testing procedures were explained to the participants and informed consent was obtained as approved by the Institutional Review Board of the University of Southern California Health Sciences Campus. Testing was conducted in the Division of Biokinesiology and Physical Therapy's Human Performance Laboratory located in the Competitive Athlete Training Zone, Pasadena, CA.

### 2.1. Procedures

Participants attended a single session during which anthropometric measurements (height, weight, and leg length) were collected and foot dominance was self-selected based on participant response to which foot they preferred to kick a ball for maximal distance. Each group completed the following battery of tests, described in detail below, in random order: LED, SLB, SLHB, and YBT. In addition, individuals performed the Vertical Jump (VJ) test to assess lower extremity strength and power.

### 2.2. Instrumentation

Reflective kinematic markers were placed on the skin over the sacrum and bilaterally on the participant's shoes at the positions best projecting the anatomical landmarks of heel and toe. Three-dimensional motion analysis was performed using a marker-based, 11-camera digital motion capturing system (250 Hz; Qualisys, Gothenburg, Sweden). Ground reaction force (GRF) data were obtained using a 1.20 × 0.60 m force plate (1500 Hz; AMTI, Newton, MA, USA) embedded into the floor surface. These data were collected synchronously using motion capture software (Qualisys Track Manger, v2.6, Gothenburg, Sweden) during the VJ and SLHB tests. The LED test system consisted of a helical compression spring (Century Springs Corp., Los Angeles, CA) mounted on a single-axis force sensor (Transducer Techniques, Temecula, CA) on a stable base with a platform affixed to the free end. The vertical component of the GRF was sampled with a data acquisition system (2000 Hz; Measurement Computing, Norton, MA) and recorded and displayed in real time with custom software.

### 2.3. Vertical Jump Test

Participants were instructed to stand adjacent to a Vertec Jump Measurement device (Sports Imports, Hilliard, OH) (positioned on the same side of their self-reported dominant hand) with their feet on the force plate shoulder width apart. After squatting to a comfortable position, they were instructed to perform a maximal vertical jump. Participants were allowed to use their arms to augment performance and they were asked to use the dominant hand to displace the highest possible horizontal swivel vane to encourage maximum jump height. Power was calculated as the product of the vertical GRF and the vertical velocity of the reflective marker placed over their sacrum using BTS SMART-Analyzer software (BTS Bioengineering, Milan, Italy). The outcome measure, peak power (W/kg, normalized to body mass (BM)), was identified for each trial and averaged across three trials for analysis.

### 2.4. Y-Balance Test

The YBT, a simplified version of the Star Excursion Balance Test, is a reliable measure of dynamic balance featuring the anterior, posterior-medial (PM), and posterior-lateral (PL) components [3]. The anterior direction is defined as directly in front of the participant and the PM and PL directions are located 135 degrees from the anterior direction, separated by 45 degrees, making the “Y” shape described in the name [3]. Participants were asked to stand and maintain balance on their dominant leg and reach as far as possible with the free limb in each direction initiating from the start position. Participants performed three trials in each direction with 40 seconds of rest between reach directions. Trials were terminated early if a participant (1) failed to maintain single-leg balance, (2) used the free limb for stance support, or (3) failed to return to the start position. Participants were provided with a visual demonstration prior to testing and tested in the following order: anterior and then PL and then PM. As the outcome measure, average distances reached in each direction as a percent of leg length (LL) were considered dependent variables for analysis (YBT_A_, YBT_PL_, and YBT_PM_, resp.). LL was measured in standing with a tape measure from the left greater trochanter to the floor.

### 2.5. Single Limb Hop and Balance Test

During the SLHB, upon verbal command, participants performed a single limb forward hop of a distance (normalized to their LL) with their dominant leg while their arms were folded across their chest. Upon landing, they were instructed to maintain single limb standing balance with arms still folded across their chest. In accordance with several groups [11, 13], the outcome measures, COP variability in the medial-lateral (ML) and anterior-posterior (AP) directions, COP_ML_ and COP_AP_, respectively, were considered dependent variables for analysis. COP excursion measurements are representative of body sway and provide information about the ability of motor system to control the COM. While all humans exhibit some level of body sway as measured by COP variability, greater COP variability has been linked to instability and falls [29, 30]. As with the previous tests, the average across three trials was used to indicate performance level.

### 2.6. Single Limb Balance Test

During the SLB, participants were asked to maintain balance on their dominant leg with their arms folded across their chest and eyes closed for a total of 15 seconds. Participants were positioned on a force plate and upon verbal command asked to lift their nondominant foot off the floor (knee bent at approximately 60°) and close their eyes. Trials were terminated early upon ground contact with the nondominant limb or when participants opened their eyes. As with the SLHB, the mean of the three trials was reported and the outcome measures of COP variability in the ML and AP directions were considered dependent variables for analysis.

### 2.7. Lower Extremity Dexterity Test

A detailed description of LED test methodology is provided in prior publications [18, 19, 27, 28]; therefore, only a brief description is provided here. Participants were positioned in an upright partially seated posture on a bicycle saddle intended to stabilize the body and minimize extraneous use of the contralateral limb and upper extremities during testing. Visual feedback was provided via computer monitor and participants were instructed to slowly compress the spring with their foot with the goal to raise the force feedback reference line as high as possible and maintain that maximal level of compression for at least ten seconds [18, 19, 27, 28]. After familiarization, at least 10 trials were performed on the self-reported dominant limb. The outcome variables, mean compression force (LED_F_) and a measure of force variability defined by the root-mean square (RMS) of the force signal during the steady-state hold (LED_RMS_), were processed using custom Matlab software (v2013b, MathWorks, Natick, MA) and were considered dependent variables for analysis.

### 2.8. Statistical Analysis

This study considered five tests and 10 total outcome measures as dependent variables detailed above: YBT (3), SLHB (2), SLB (2), LED (2), and VJ (1). Principal components analysis (PCA) was performed to identify the best linear fit to the data using a series of perpendicular vectors or principal components (PCs) [31]. Within each PC vector (i.e., column), the structure of the correlations and nonzero numerical values in each column quantify the relative positive or negative correlations among variables [31]. To put it simply, we used PCA as a method of examining the contributions of the outcomes measures to balance and the associations among the outcome measures. Due to the differences in units and normal distributions among variables, and for comparison purposes, we calculated the standard score (*z*-score) of each variable and used their standardized normal distribution values as the PCA dataset [32]. The PCs are presented in descending order quantifying their contributions to balance such that the first principal component explained the largest amount of variance. We note that the first five PCs captured at least 80% of the total variance; therefore, we limited our analysis to them: first five PCs. SPSS version 22 (IBM, Armonk, NY) and Matlab were used for this analysis and the significance level was set at *p* ≤ 0.05.

## 3. Results

The means and standard deviations of all dependent variables are presented in Table 1. Outcome measures on all of the tests, by all subjects, were within normal ranges when compared to previously published data [3, 12, 18, 33, 34]. Our PCA data are presented in numerical form (Table 2). Loading values quantify the strength and direction of the relationships between variables and range between −1 and 1, where 1 is total positive correlation, 0 is no correlation, and −1 is total negative correlation.

**Figure 1:**
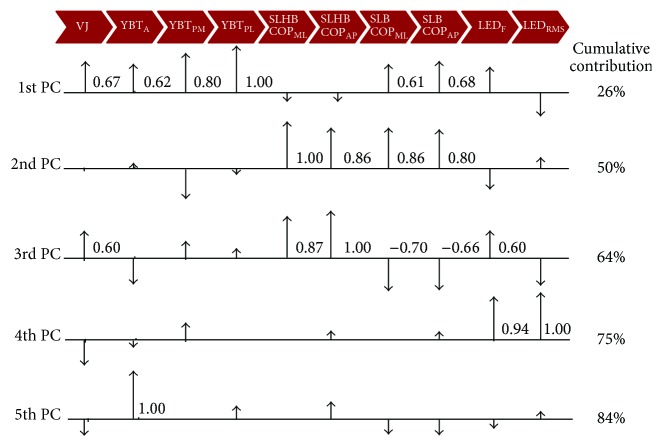
Visualization of PC loadings. The scaled metric loadings for the first five PCs are illustrated above. All loadings are shown, but numerical values are only listed if they are ≥±0.60. The signs of the loadings are indicated by the direction of the arrowheads.

The 1st PC explained 26% of the total variance in balance with the highest loadings assigned to YBT_PL_ and YBT_PM_ (1.00 and 0.80, resp.). Furthermore, we report additional moderate, positive correlations between VJ, YBT_A_, and SLB COP_AP_ and COP_ML_ with loading values ranging from 0.68 to 0.61. The 2nd PC explained an additional 24% of the variance with all SLHB and SLB COP variables exhibiting the highest loadings (1.00–0.80, resp.). In the 3rd PC, the SLHB COP measures featured the highest loadings, explaining 14% of the variance. Interestingly, while the relationships between SLHB and SLB COP variables were moderate to strong in both the 2nd and 3rd PCs, they were negatively correlated in the 3rd PC (−0.62 and −0.59), unlike the 2nd, which featured positive correlations. In addition to the disambiguation between static (SLB) and dynamic (SLHB) balance variables we report in the 3rd PC, we further note that LED_F_ showed a moderate positive association with SLHB variables while LED_RMS_ was positively correlated with SLB variability. We further report moderate positive correlations with VJ and LED_F_. The 4th PC explained an additional 11% of the variance in balance and revealed that the LED variables were highly positively correlated (1.00 and 0.94, resp.) with each other and no other metric. Finally, YBT_A_ solely dominated the 5 PCs and explained 9% of the total variance. In order to further highlight our results, we provided a visual representation of the respective loadings for each of the first five PCs, first presented in Table 2, in Figure 1.

## 4. Discussion

This is the first study, to our knowledge, to investigate the relationship among multiple balance tests and outcome measures traditionally used to assess balance in young individuals. The battery of measures examined in this study represent a range of static and dynamic tests that are commonly used to assess balance in healthy individuals or following lower extremity injury or to identify those at greater risk for injury [1, 3, 5–7, 9, 12, 14, 29, 30, 35, 36]. The combination of measures of static and dynamic balance, strength, and sensorimotor processing considered in this study allowed the unique opportunity to explore the relationships between the numerous components that we speculate to contribute to overall balance. Understanding the relationships and hierarchy among outcome measures in young healthy individuals using PCA provides some insight into the contributors to balance. In this paper, we present our PCA data in two distinct formats, numerically (Table 2) and graphically (Figure 1). For ease of comparison, we ordered the measures on a continuum from what can be considered more dynamic (YBT) to more static (SLB) balance tests anchored at the extremes by the outcome measures most associated with strength (VJ) and sensorimotor processing (LED) (top to bottom, Tables 1 and 2; left to right, Figure 1). When considered together, 84% of the variance in balance was explained by the first five PCs with each individually contributing to 9–26% of the total variance. The 6th and further PCs each contribute to relatively small percentages (<9%) of total variance and were not considered in our analysis due to the potential for overinterpretation.

Our analysis indicated that balance is best distinguished by a combination of outcome measures from both static and dynamic tests as the SLB and Y-balance tests were the most heavily loaded in the 1st PC. Together, these measures explained 26% of the total variance in balance. YBT_PL_ featured the highest loading and revealed strong and moderate positive relationships with YBT_PM_ and YBT_A_, respectively. Multiple studies have reported correlations between lower limb strength [2, 17], range of motion [37, 38], and Y-balance performance in all three directions. Therefore, it is not surprising that there was also a moderate positive correlation with VJ, a widely accepted estimate of leg power and strength [33, 39, 40]. The inclusion of these measures in the 1st PC suggests that the multijoint coordination and strength required to perform more dynamic tests are important contributors to balance. However, the presence of moderate positive correlations with SLB variability (COP_ML_ and COP_AP_), the most static balance test, suggests that the detection and correction of smaller perturbations are also important to balance ability. Measurements of COP variability during SLB tests are validated methods of quantifying what is referred to as static balance or stability [1, 29, 34]. Relatively small displacements of the lower limb, particularly at the ankle, are used to maintain balance and are reflected in COP variability [15]. The presence of the SLB variables in the 1st PC seems to indicate moderate dependence on sensory inputs for detection of small perturbations while maintaining balance.

After considering the contribution of these measures to balance, an additional 24% of the variance was explained by grouping of COP variables during both SLHB and SLB in the 2nd PC. It is not surprising that these variables were strongly associated as both are measures of COP variability, which are representative of modulation of ML and AP COP by the motor system. While the mean values for SLHB variability were slightly, although, we emphasize not significantly, greater than the SLB (Table 1), we concede that is due to the more dynamic nature and slightly increased strength demands of the SLHB. When taken together, however, the correlations among the outcome measures from static and dynamic balance tasks support prior research that reported no differences performance on both static and dynamic postural control tasks [29]. Strong positive correlations among these variables suggest that both small and large corrective actions during static and dynamic tests are important overall contributors to balance. Moreover, the negative correlation to YBT_PM_ supports our speculation that COP variables are indicative of separate contributions to balance compared with what is measured during more dynamic, multijoint coordination-, and strength-driven tasks.

In the 3rd PC, which further explained 13% of the total variance, COP velocities in the AP and ML directions during the SLHB were again the leading contributors. Interestingly, in this PC, SLHB measures were moderately negatively correlated with SLB measures, unlike the 2nd PC. The contrasting relationships between COP variables during SLB and SLHB observed between the PCs, as well as the slight differences in mean performance values presented in Table 1, support the notion that COP variability in these two tasks represents similar but distinct mechanisms of balance [1, 4, 12, 14, 30, 36, 41]. The SLHB is a standard objective measure often used to evaluate dynamic balance following training protocols and when examining patients following lower limb injury or surgery [1, 7, 9, 13]. While static balance measures are of clinical relevance, in terms of function, emphasis is often placed on dynamic balance tests (e.g., SLHB and YBT) because they are more representative of activities of daily living (ADLs) and have greater sensorimotor demands. To limit the potential influence of strength and distance hopped on performance of this test, we asked participants to hop a standardized distance equal to the length of their lower limb. The characterization of the SLHB as a more dynamic measure of balance than the SLB is further supported by the moderate positive relationship with VJ. Moreover, the weak and discordant relationship with YBT variables could support the argument that the SLHB is less dynamic than the Y-balance protocol and results in smaller perturbations to the COM within the BOS.

We find it particularly noteworthy that, in the 3rd PC, LED* compression force* (LED_F_) was positively correlated with* dynamic* balance variables (SLHB) while LED* force variability* (LED_RMS_) was more closely associated with* static* balance variables (SLB). The dependent variable for the LED test has traditionally been the average of the three hold phases with the highest mean compression force (LED_F_). This is because the spring becomes increasingly unstable as it is compressed further. Thus, the level of maximal sustained spring compression is informative of the maximal instability that can be controlled by the isolated leg. The springs are designed to reach these high levels of instabilities at very low forces (ca. 150 N for the leg or ca. 10% of body weight). The LED_F_ has shown to be sensitive to sex differences [18, 28] and age effects [18] and correlate well with whole-body agility [27]. More recently, LED_F_ has shown strong correlations with single limb cross-country ski distance, which one can easily argue is a* dynamic* measure, but showed no correlation with a* static* single limb balance test [42]. Additionally, the force fluctuations (e.g., RMS) during the hold phases of the SD paradigm for the upper extremity were first introduced as a method of quantifying differences in performance (i.e., sensorimotor processing) attributed to several clinical conditions [18, 22, 23]. Greater RMS indicates larger dynamical dispersion and suggests weaker (or looser) corrective actions by the neuromuscular controller enforcing the sustained compression. Now, in this study, we include force fluctuations during the LED test (LED_RMS_) as a complementary, but equally important, measure of sensorimotor processing of the lower limb in healthy individuals.

The 4th PC accounted for 11% of the total variance in balance. Strong and positive relationships between both LED variables (LED_F_ and LED_RMS_) were noted in this PC, suggesting that the sensorimotor control may uniquely contribute to balance. These results complement previous studies, including numerous of our own featuring the SD paradigm for the fingers, which have found that sensorimotor processing during dexterous tasks (e.g., dexterity) represents a different functional domain than strength or whole-arm coordination [18–21, 24–27, 43, 44]. While no correlations greater than 0.60 were noted with variables of other tests in this PC, LED variables were negatively correlated to VJ (−0.54), a measure of lower extremity strength and power, which further complements our prior work suggesting that lower extremity dexterity is independent of strength [19]. In the 5th PC, YBT_A_ was the sole contributing variable to the 9% of the total variance explained. While the relative contribution to overall variance explained is comparatively small, the fact that YBT_A_ shows no correlation with the other YBT variables implies it may represent a different functional dimension than the posterior YBT directions. The anterior direction can be considered primarily uniplanar, whereas the PM and PL directions clearly require coordination of multiple joints across multiple planes. This is also supported by the data in the 1st PC that show strong correlations between the YBT PM and PL directions and only a moderate correlation with the anterior direction and again in the 3rd PC, where YBT_A_ shows weak negative correlations with the YBT posterior directions.

The results presented in this study speak to the fact that balance is dependent on multiple contributors. We find that the outcome measures of tests can be thought of as quantifying the strength, multijoint coordination, dynamic and static stability, and sensorimotor processing contributors to balance—which we find cannot be assessed independently and simultaneously by any one single outcome measure. This makes it difficult to truly understand the sensorimotor mechanisms of balance, let alone the effects of lower extremity injury on balance ability. This may begin to explain why there are conflicting reports of effects of injury on outcome measures of balance tests or effectiveness of training or rehabilitation protocols for improving these measures. For example, while several studies report differences between control and clinical groups in some or all measures associated with SLB tests [5, 14, 15, 17, 30], others report no differences between or within groups. Previous authors suggest that the inconsistent reports may be attributed to the fact that the SLB test loses sensitivity over the time course of recovery and is not challenging enough to be truly representative of sports-related activities, where balance deficits become more apparent [37, 45, 46]. There are also similar conflicting reports across more dynamic balance tests including the YBT. Multiple groups have reported significant differences between side-to-side YBT outcome measures (e.g., functional reach distances) in participants with chronic ankle instability (CAI) [37, 45]. However, one reported side-to-side differences in participants with CAI, but no group differences between healthy participants and those with CAI [45]. The inconsistencies in the literature in terms of success of both static and dynamic balance tests in the clinic support our hypothesis that these measures provide informative, yet limited, information about the mechanisms of balance ability. It is important to point out that our study was conducted in recreationally active young adults with no recent lower extremity injuries. Our results compel future studies in clinical populations to develop and assess the ability of outcome measures to gauge the efficacy of rehabilitation regimens for lower extremity injuries, including, but not limited to, CAI and ACL tears.

We successfully identified distinct relationships among outcome measures that suggest they together reveal latent functional contributors to balance. After considering the origin, nature, and use of each outcome measure, we propose that the latent contributors to balance they reveal are those of strength, multijoint coordination, and sensorimotor processing. They represent distinct functional domains, which are revealed by the relationships among the loadings in our PCA results. The multiple strong to moderate correlations (loadings) in the 1st PC suggest that a combination of strength, multijoint coordination, and static stability (i.e., detection of small perturbations from the sensory system) is the leading contributors to balance. However, in the subsequent PCs, other contributors gain prominence. The 2nd PC placed strong emphasis on a combination of static and dynamic balance variability. The fact that they are not strongly correlated with the other outcome measures strengthens our assertion that both static and dynamic balance are similar functional features that are distinct from strength or multijoint coordination. These results indicate that the combined corrective actions by the motor system during both static and dynamic balance tests are important contributors to balance. While the SLB and SLHB tests have similar origins and functional features, there are differences that warrant consideration. The more dynamic nature of the SLHB naturally leads one to assume that there would be different strength and coordination requirements, which is supported by the negative correlations with SLB variables and positive correlation with VJ revealed in the 3rd PC. The opposite loading signs of the SLHB in the 2nd and 3rd PCs speak to the fact that it may be informative of both static and dynamic balance, but the moderate correlation with VJ emphasizes that dynamic stability should be considered in the context of submaximal force performance to reduce the influence of strength, which, as we mentioned previously, can dilute the information gleaned from such dynamic outcome measures. Additionally, the correlations we report between the LED test variables and COP variability during both SLB and SLHB indicate that the LED test may be a useful tool to quantify sensorimotor processing during both static and dynamic balance measures. Finally, our analysis further indicated that sensorimotor processing, as quantified by the LED test, was another distinct contributor to balance (4th PC) that also tended to be independent of strength. This confirms our prior work for both the upper and lower extremity [18–20, 24, 27, 28, 43, 44] and mirrors work about the development of dexterity in children where the SD test was seen as a functional dimension distinct from strength and whole-arm coordination [26]. These results in lower extremity function also complement our findings in the upper extremity [47] despite the obvious evolutionary, anatomical, and functional differences and suggest fundamental, body-wide mechanisms for function. We do acknowledge, however, that sensory or motor constructs (e.g., proprioception, vision, and motor control) were not specifically quantified in this study. We also note that these data represent balance ability in healthy individuals. It is not clear how these results would change if individuals with sensory or motor deficits were included.

Our results support the well-accepted notion that balance is a complex, albeit everyday, task but provide a quantitative context within which to understand its contributors. Thus, we lend evidence to the idea that depending on a single outcome measure to quantify balance, its deficits, and its rehabilitation is arguably deficient. We recommend using a combination of complementary assessments to quantify its multiple contributors: strength, multijoint coordination, stability (both static and dynamic), and sensorimotor processing. This will not only improve assessment accuracy on an individual level, but also facilitate the development of customized rehabilitation or training regimens to target improvements of individual contributors deemed deficient or in most need of attention. Furthermore, the ability of the novel LED paradigm to successfully quantify sensorimotor processing, in addition to the correlations with both static and dynamic balance measures reported in this study, makes it a useful tool to quantify and promote that specific contributor. Thus, it complements the other well-accepted measures of strength and multijoint coordination currently in use in both research and clinical settings. Note that because the LED test requires very low forces and tests the isolated leg while the hip and torso are held steady, it is particularly well suited to clinical, postsurgical, and postinjury populations who cannot perform other outcome measures mostly geared towards healthy athletic young adults.

## Figures and Tables

**Table 1:** Mean performance data from all subjects.

Metric	Variable	Mean ± SD
VJ	Power (W/kg, % BM)	48.1 ± 9.6
YBT	YBT_A_ (% LL)	63.4 ± 4.8
YBT	YBT_PM_ (% LL)	106.6 ± 11.3
YBT	YBT_PL_ (% LL)	102.4 ± 10.1
SLHB	COP_ML_ (mm/s)	0.03 ± 0.01
SLHB	COP_AP_ (mm/s)	0.03 ± 0.01
SLB	COP_ML_ (mm/s)	0.02 ± 0.01
SLB	COP_AP_ (mm/s)	0.01 ± 0.003
LED	LED_F_ (N)	130.7 ± 13.4
LED	LED_RMS_ (N/s)	0.08 ± 0.03

**Table 2:** Principle component loadings.

Variable	1st PC	2nd PC	3rd PC	4th PC
VJ	*0.67*	−0.03	*0.60*	−0.54
YBT_A_	*0.62*	0.07	−0.52	−0.15
YBT_PM_	*0.80*	−0.50	0.40	0.41
YBT_PL_	**1.00**	−0.06	0.23	0.04
SLHS COP_ML_	−0.19	**1.00**	*0.87*	0.03
SLHS COP_AP_	−0.18	*0.86*	**1.00**	0.20
SLS COP_ML_	*0.61*	*0.86*	*−0.70*	0.04
SLS COP_AP_	*0.68*	*0.80*	*−0.66*	0.17
LED_F_	0.52	−0.37	*0.60*	*0.94*
LED_RMS_	−0.50	0.18	−0.57	**1.00**

% Contribution	**26.07%**	**23.53%**	**14.57%**	**10.49%**
Cumulative	**26.07%**	**49.59%**	**64.17%**	**74.66%**

Normalized loadings for ease of comparison; italics font in each column indicates (≥0.60) positive and negative correlations, respectively, with the dominant variable in bold.

## Outcome Measure Abbreviations

VJ: Vertical Jump test
YBT: Y-Balance test
SLHB: Single Limb Hop and Balance test
SLB: Single Limb Balance test
LED: Lower Extremity Dexterity test.
